# Impact of five years of peer-mediated interventions on sexual behavior and sexually transmitted infections among female sex workers in Mombasa, Kenya

**DOI:** 10.1186/1471-2458-8-143

**Published:** 2008-04-29

**Authors:** Stanley Luchters, Matthew F Chersich, Agnes Rinyiru, Mary-Stella Barasa, Nzioki King'ola, Kishorchandra Mandaliya, Wilkister Bosire, Sam Wambugu, Peter Mwarogo, Marleen Temmerman

**Affiliations:** 1 International Centre for Reproductive Health, Mombasa, Kenya; 2 International Centre for Reproductive Health, Department of Obstetrics and Gynecology, Ghent University, Belgium; 3 Family Health International, Nairobi, Kenya; 4 Coast Provincial General Hospital, Mombasa, Kenya

## Abstract

**Background:**

Since 2000, peer-mediated interventions among female sex workers (FSW) in Mombasa Kenya have promoted behavioural change through improving knowledge, attitudes and awareness of HIV serostatus, and aimed to prevent HIV and other sexually transmitted infection (STI) by facilitating early STI treatment. Impact of these interventions was evaluated among those who attended peer education and at the FSW population level.

**Methods:**

A pre-intervention survey in 2000, recruited 503 FSW using snowball sampling. Thereafter, peer educators provided STI/HIV education, condoms, and facilitated HIV testing, treatment and care services. In 2005, data were collected using identical survey methods, allowing comparison with historical controls, and between FSW who had or had not received peer interventions.

**Results:**

Over five years, sex work became predominately a full-time activity, with increased mean sexual partners (2.8 versus 4.9/week; *P *< 0.001). Consistent condom use with clients increased from 28.8% (145/503) to 70.4% (356/506; *P *< 0.001) as well as the likelihood of refusing clients who were unwilling to use condoms (OR = 4.9, 95%CI = 3.7–6.6). In 2005, FSW who received peer interventions (28.7%, 145/506), had more consistent condom use with clients compared with unexposed FSW (86.2% versus 64.0%; AOR = 3.6, 95%CI = 2.1–6.1). These differences were larger among FSW with greater peer-intervention exposure. HIV prevalence was 25% (17/69) in FSW attending ≥ 4 peer-education sessions, compared with 34% (25/73) in those attending 1–3 sessions (P = 0.21). Overall HIV prevalence was 30.6 (151/493) in 2000 and 33.3% (166/498) in 2005 (*P *= 0.36).

**Conclusion:**

Peer-mediated interventions were associated with an increase in protected sex. Though peer-mediated interventions remain important, higher coverage is needed and more efficacious interventions to reduce overall vulnerability and risk.

## Background

Despite 25 years of HIV prevention, in many settings HIV incidence remains high in the general population and especially among most-at-risk groups [1]. In particular, women in sub-Saharan Africa remain disproportionately affected by HIV, which reflects and reinforces underlying gender inequities. Female sex workers (FSW), estimated to number tens of millions worldwide [2], are highly vulnerable to acquiring and transmitting sexually-transmitted infections (STI) including HIV [3]. Targeting most-at-risk groups such as FSW is a key strategy for preventing HIV in both concentrated and generalized HIV epidemics [2,4,3].

Preventing HIV among FSW requires implementation of evidence-based interventions, adapted to local circumstances [5,6]. Data supports the effectiveness of FSW interventions which address the conditions and context of sex work, including targeted condom promotion and distribution; skills development (such as condom negotiation); STI/HIV education; and STI treatment [7-13]. Though several studies have shown peer-educators can effectively deliver these interventions [3,14-16], additional evidence is needed of the impact of outreach and peer-networks in diverse settings, and sustainability over longer periods of time. Using a before-after design with a baseline cross-sectional survey in 2000 and repeated in 2005, we aimed to evaluate the impact of five years of peer-mediated STI/HIV prevention interventions among FSW in Mombasa, Kenya. Historical and internal controls are compared to evaluate changes in HIV-related knowledge and attitudes, behavior, and STI/HIV prevalence.

## Methods

### Setting and population

Mombasa, in Kenya's Coast province, is a major economic centre in the region, with important tourism, port, rail and industrial enterprises, as well as a large FSW population. The study took place in a division of Mombasa, Kisauni, which has around 250 000 inhabitants, 70 000 households and a population density of 2 278 inhabitants/km^2 ^[17]. More than half the population of Kenya lives on less than $2 a day and the Gross Domestic Product per person is an estimated $547 [18]. The population studied consisted of FSW, defined as any woman who reported having received money or gifts in exchange for sex in the past year. Sex workers are either full- or part-time, and work from bars, hotels, streets and homes. They frequently also are involved in other small businesses, including selling foodstuffs, vegetables, and in some areas, local brew on the roadside. Commonly the clients of sex workers are employed at local factories or are *matatu *(minibus taxi) touts [19].

### Intervention project

Between 2000 and 2005, the International Centre for Reproductive Health in collaboration with Family Health International (FHI) implemented a peer-mediated FSW intervention in Mombasa (IMPACT project). This aimed to reduce unprotected sex acts by increasing condom use and reducing the number of sexual partners; and secondly to empower FSW to control their working and social lives by increasing their knowledge of STI/HIV and condom negotiation skills. Finally it aimed to reduce co-factors associated with HIV transmission by providing information on, and referrals for STI treatment, and HIV testing and counseling.

Sixty-two FSW from Kisauni division were selected and trained as peer educators in 2000; 57 of whom were retained throughout the five-year period. Peer educators were selected through key informants (bar maids, patrons) at identified hotspots in the area. Study staff aimed to select FSW who were willing to be a peer leader, had a substantial network of peers, were likely to remain in the area for an extended period, and had some knowledge of the key topics. Those selected, attended a five-day training course on STI/HIV signs and symptoms; STI/HIV prevention and treatment; promotion and distribution of male and female condoms; and teaching of safe sex negotiation skills. Six-day advanced and three-day refresher training was provided midway in the project. Peer educators acted as links between the local FSW community and the project, facilitating local involvement and participation. They conducted one-on-one or weekly-group sessions, mostly in the houses of FSW or at a drop-in centre based within the community. Besides functioning as training and meeting facility, the drop-in centre was used for distributing information, education and communication (IEC) materials and condoms, and for voluntary counselling and testing (VCT) services. Diverse mediums were used to transfer knowledge and health promotion messaging, such as peer-mediated drama, role playing exercises, use of picture codes (visual images used for engaging discussion on sensitive topics) and video sessions. Peer-led activities occurred throughout the five-year period at a relatively constant rate. Peer educators also led monthly community gatherings with active participation of FSW, youth and other community members. These provided HIV education, condom promotion and other risk-reduction activities and were accompanied by mobile VCT services, facilitating entry to HIV testing. A field coordinator updated peer educators on new developments in HIV prevention and regularly attended peer-education sessions to monitor the accuracy of information given and to assist in responding to questions.

### Study design

Over a two-month period, in February-March 2000 and October-November 2005, we conducted pre- and post-intervention cross-sectional surveys. To enhance comparison, the repeat survey adhered to the design and methodology of the pre-intervention survey, detailed previously [20]. In brief, initial respondents (seeds) were identified from bars, guest houses and the street, with subsequent participants recruited using snowball sampling. To limit potential for friendship bias, we restricted the maximum number of women recruited through one participant to 10. Eligible participants were self-reported FSW, older than 16 years and working within Kisauni. Peer educators were excluded from participation in the repeat survey.

Study procedures were performed by qualified staff at the drop-in centre. These procedures included structured questionnaires; VCT; collection of blood plasma and urine samples; and gynecological examination, with speculum insertion and collection of endocervical and high vaginal swabs. Where indicated, FSW received STI treatment as per the Kenya STI guidelines free of charge. Women were encouraged to learn their HIV status and offered same day HIV testing and counseling, using on-site serial testing. Those who tested positive for HIV were referred to a comprehensive HIV care clinic where antiretroviral treatment is provided at no cost for those requiring it.

### Laboratory investigations

Investigations for HIV, syphilis, *Neisseria gonorrhoeae *and *Chlamydia trachomatis *were performed in 2000, with methods previously described [20]. In the repeat survey, parallel rapid HIV testing was done in the laboratory with Determine™ HIV-1/2 (Abbott Laboratories by Abbott Japan co Ltd, Minato-Ku, Tokyo, Japan) and Uni-Gold™ HIV (Trinity Biotech plc, Bray, Ireland). For five discordant HIV results, an enzyme-linked immunosorbent assay was performed as tie-breaker (all five were HIV seronegative). A rapid plasma reagin test (Human GmbH, Weisbaden, Germany) was used for syphilis screening. Endocervical secretions were tested for gonococcus using gram stain and culture with blood agar (International Diagnostic Group, Lancashire, United Kingdom). Chlamydia was not analyzed in the 2005 survey due to financial constraints, but additional tests were performed, including a wet mount preparation to identify candida and *Trichomonas vaginalis*, as well as a gram stain to detect bacterial vaginosis (diagnosed with Nugent's criteria).

### Data management and statistical analysis

Data were double entered by separate clerks. Following data checking and cleaning, Intercooled Stata 8.0 (Stata Corporation, College Station, Texas, USA) was used for statistical analysis. A standard WHO framework was used to assess risk measures within three categories: unprotected sex (number of partners and condom use); empowerment of sex workers (knowledge and condom negotiation); and HIV transmission efficiency (when condoms fail or are not used) [5]. Reproductive tract infections (RTI) such as syphilis and gonococcus were considered important co-factors of HIV transmission efficiency and analyzed within this category. This strategic framework provides a systematic means of assessing desired outcomes of HIV prevention services for FSW. Sexual partners of FSW were categorized as: emotional partners (husband/boyfriend) or clients (regular clients and casual one-time clients).

Analysis included descriptive statistics of the distribution and central values of socio-demographic and sex work characteristics, and indicators of sexual behavior. Significance testing was done to compare differences in participants' characteristics using chi-square and unpaired Student's *t *tests.

To evaluate overall effects of the project (assuming that peer-mediated interventions influence norms in the whole population), we compared outcomes in the 2005 sample of FSW with those in 2000. Odds ratio's (OR) with 95% confidence intervals (CI) were calculated.

Using the 2005 data set, effects of individual-level exposure to peer interventions were determined by comparing women who had ever attended a peer-education session with those who had not. To determine whether the number of peer-education sessions attended had an effect on outcomes, we compared women who had attended four or more peer education sessions in the past six months with those who had attended fewer sessions. Logistic regression models were constructed to control for potential confounding, giving adjusted odds ratios (AOR) of the association between exposure to peer-mediated services and outcome variables. Age, marital status, education and place of work were included in multivariable models.

### Ethical approval

Written informed consent was obtained from all participants before study entry. For both surveys, ethical approval was obtained from the Kenyan national ethics committee at Kenyatta National Hospital (Ethics and Research Committee) and from the Protection of Human Subjects Committee of FHI, USA.

## Results

The number of study participants was 503 in 2000 and 506 in 2005. Comparing the population in 2000 and 2005, relatively small differences were seen in age and income (Table 1). Larger changes had occurred in marital status, proportion of women in full-time sex work and workplace (from homes and guest-houses in 2000 to mainly bars and nightclubs in 2005). While the proportion of single women remained unchanged, in 2000 a substantial proportion (39.4%) were married or cohabiting, compared with only 2.5% in 2005. Sex work had changed from a predominately part-time to full-time activity; women with an alternative source of income decreased from 67.0% to 37.7% (P < 0.001). Most women in 2005 reported usually receiving payment for sex with cash (86.6%; 438/506), with the remainder receiving a combination of cash and gifts, clothing or food. In both time periods, about 90% of FSW had one or more children.

**Table 1:** Participant's socio-demographic and socio-economic characteristics

	**N**	**2000 survey**	**N**	**2005 survey**	***P*-value**^¥^
Age, mean years (sd)	503	31.0 (8.8)	504	29.5 (7.5)	0.004
Marital status, % (number of women)	503		506		
Single		43.9% (221)		43.1% (218)	
Married/cohabiting		39.4% (198)		2.8% (14)	
Separated/divorced		15.1% (76)		46.6% (236)	
Widowed		1.6% (8)		7.5% (38)	<0.001
Education, % (number of women)	502		506		
None		19.3% (97)		10.1% (51)	
Primary level		60.0% (301)		60.3% (305)	
Secondary level		18.5% (93)		27.1% (137)	
Tertiary level		2.2% (11)		2.6% (13)	< 0.001
Religion, % (number of women)	502		506		
Catholic		17.1% (86)		28.9% (146)	
Muslim		55.2% (277)		32.4% (164)	
Protestant		26.7% (134)		37.2% (188)	
Other		1.0% (5)		1.6% (8)	< 0.001
Number of living children, % (number of women)	503		506		
0		7.6% (38)		12.9% (65)	
1		22.9% (115)		28.1% (142)	
2–3		38.0% (191)		41.5% (210)	
> 3		31.6% (159)		17.6% (89)	< 0.001
Weekly income from sex work alone, mean USD (sd)	503	19.9 (16.9)	506	22.5 (15.8)	0.01
Alternative source of income, % (number of women)	503		506		
None		33.0% (166)		62.3% (315)	
Formal employment		1.8% (9)		1.4% (7)	
Informal trade		61.6% (310)		31.6% (160)	
Other		3.6% (18)		4.7% (24)	< 0.001
Where clients usually obtained, % (number)	502		506		
Home		47.8% (240)		15.9% (81)	
Hotel/guest house		40.0% (201)		0.8% (4)	
Bar/nightclub		9.4% (47)		75.5% (382)	
Street/beach		2.2% (11)		7.5% (38)	
Other		0.6% (3)		0.2% (1)	< 0.001

^¥ ^*P*-value was calculated using chi-square test for categorical and unpaired Student's *t *test for continuous variables. Exchange rate of 1USD: 74 Kenya Shillings

In the 2005 survey, 28.7% (145/506) reported having attended peer-mediated interventions at least once. In the past six months, 78.6% (114/145) of these women had attended peer-education sessions, a median of four times (IQR 2–7). Eighty four percent of FSW who attended peer education had one-on-one sessions with peer educators (122/145). In a multiple-response question, women reported having participated in peer-mediated drama (43%; 62/145), role plays (41%; 59/145), picture code (32%; 47/145) and video sessions (3%; 4/145). About half (55%; 80/145) the women who received peer interventions reported that a peer educator had referred them to HIV testing.

### Population-level effects, comparing female sex workers in 2000 and 2005

#### Unprotected sex

With more women reliant solely on sex work for their income in 2005, the mean number of sexual partners increased from 2.8 to 4.9 per week (*P *< 0.001). Merely 7.0% of women had four or more one-time clients per week in 2000; this increased to 33.2% in 2005 (OR = 6.6, 95%CI = 4.4–10.2; *P *< 0.001; Table 2). These changes were accompanied by marked increase in condom use with clients, in last sex act (47.3% versus 85.8%; *P *< 0.001) as well as in consistent use, from 28.8% to 70.4% (OR = 5.9, 95%CI = 4.4–7.8; *P *< 0.001). No improvements were achieved with emotional partners, where still 80% of women did not use condoms consistently.

**Table 2:** Sexual behavior and reproductive tract infections among female sex workers in 2000 and 2005, and a comparison between those in 2005 who had or had not received peer-mediated interventions

	**2000 survey**	**2005 survey**	OR*(95%CI)	**Non peers**	**Peers**	AOR** (95%CI)
**UNPROTECTED SEX**						

Total years in sex work, mean (sd)	9.2 (7.8)	5.6 (7.0)	-	5.5 (4.7)	6.1 (5.1)	-
≥ 4 sexual partners in past week	29.2% (147/503)	48.0% (243/506)	2.2 (1.7–2.9)	50.7% (183/361)	41.4% (60/145)	0.8 (0.5–1.1)
≥ 4 casual one-time clients in past week^§^	7.0% (33/472)	33.2% (148/446)	6.6 (4.4–10.2)	35.9% (115/320)	26.2% (33/126)	0.7 (0.5–1.2)
Used a condom with last client	47.3% (238/503)	85.8% (434/506)	6.7 (4.9–9.2)	82.6% (298/361)	93.8% (136/145)	3.5 (1.6–7.4)
Always used a condom with clients	28.8% (145/503)	70.4% (356/506)	5.9 (4.4–7.8)	64.0% (231/361)	86.2% (125/145)	3.6 (2.1–6.1)
Always used a condom with boyfriend/husband^€^	20.3% (66/325)	20.1% (60/298)	1.0 (0.7–1.5)	17.6% (37/210)	26.7% (24/90)	1.7 (0.9–3.1)

**SEX WORKER EMPOWERMENT**						

Own idea to use the condom at last sex	89.9% (214/238)	90.3% (392/434)	1.0 (0.6–1.8)	88.3% (263/298)	94.9% (129/136)	2.3 (1.0–5.5)
Provided the condom at last sex herself	72.7% (173/238)	71.2% (309/434)	0.9 (0.6–1.3)	68.5% (204/298)	77.2% (105/136)	1.6 (1.0–2.6)
HIV-related knowledge and attitudes						
Thinks a client might be HIV-infected	22.1% (111/502)	30.6% (154/504)	1.5 (1.2–2.1)	30.0% (108/360)	31.3% (45/144)	1.2 (0.8–1.8)
Knows people with HIV can look healthy	95.6% (481/503)	94.2% (474/503)	0.7 (0.4–1.4)	92.8% (334/360)	97.2% (141/145)	3.0 (1.0–9.1)
Mentions advantage to knowing HIV status	78.3% (393/503)	94.4% (475/503)	4.7 (3.0–7.6)	93.0% (334/359)	97.9% (142/145)	3.2 (0.9–11.3)
Knows ≥ 2 symptoms of STI in women	81.9% (412/503)	66.4% (336/506)	0.4 (0.3–0.6)	61.2% (221/361)	79.3% (115/145)	2.7 (1.6–4.3)
Cited ≥ 2 ways to prevent STI	43.9% (221/503)	30.4% (154/506)	0.6 (0.4–0.7)	23.8% (86/361)	46.9% (68/145)	2.8 (1.9–4.3)
Ever refused client unwilling to use condoms	41.4% (208/503)	77.7% (393/506)	4.9 (3.7–6.6)	75.4% (272/361)	83.5% (121/145)	1.7 (1.0–2.8)

**TRANSMISSION EFFICIENCY**						

Awareness of own HIV status	5.2% (26/503)	40.2% (203/505)	12.3 (7.9–19.8)	38.2% (138/361)	44.8% (65/145)	1.4 (0.9–2.1)
Self reported history of RTI in past year						
Excessive or foul smelling vaginal discharge	20.9% (105/503)	47.2% (239/506)	3.4 (2.5–4.5)	48.8% (176/360)	43.5% (63/145)	0.8 (0.5–1.2)
Genital ulcer	11.1% (56/503)	19.6% (99/505)	1.9 (1.3–2.8)	21.1% (76/361)	16.6% (24/145)	0.8 (0.5–1.3)
RTI prevalence						
Syphilis infection	2.0% (10/500)	2.0% (10/498)	1.0 (0.4–2.4)	2.5% (9/235)	0.7% (1/142)	0.3 (0.04–2.3)
Chlamydial infection	4.2% (21/502)	-	-	-	-	-
Gonorrhoeal infection	1.8% (9/502)	1.0% (5/484)	0.6 (0.1–1.9)	1.2% (4/432)	0.7% (1/142)	0.6 (0.7–5.9)
Trichomonas vaginalis infection	-	20.4% (100/490)	-	21.8% (76/348)	16.9% (24/142)	0.7 (0.5–1.2)
Bacterial vaginosis infection	-	46.5% (205/441)	-	47.8% (149/312)	43.4% (56/129)	0.9 (0.6–1.3)
Candida infection	-	22.4% (110/491)	-	21.0% (73/348)	25.9% (37/143)	1.5 (0.9–2.3)

Notes to Table 2:

RTI reproductive-tract infection. Cells contain %, n/N unless indicated.

^§^Among those women having had a one-time client in the past week

^€^Among those women who reported having a boyfriend/husband

*H_0_:odds ratio = 1

**AOR, odds ratio adjusted for age, marital status, education and place of work. Logistic regression was used to calculate AOR.

#### Sex worker empowerment

Among women reporting to have used a condom during last sex with a paying client, approximately 90% in both surveys mentioned this was their idea, and around 70% provided the condom themselves. The proportion of women who mentioned there is an advantage to knowing once HIV status was higher in 2005 (94.4% versus 78.3%; *P *< 0.001). An increase was also seen in the proportion of women who had ever refused one or more clients unwilling to use condoms from 41.4% to 77.7% (OR = 4.9, 95%CI = 3.7–6.6; *P *< 0.001). Still, of the 150 women in 2005 who reported inconsistent condom use with clients, 93 (62.0%) cited 'client refusal' as the reason for inconsistent use.

#### Reduced transmission efficiency

Being aware of HIV status increased markedly from 5.2% in 2000 to 40.2% in 2005. Syphilis prevalence remained unchanged, though increases had occurred in history of genital ulcer disease or abnormal vaginal discharge.

### Individual-level effects, comparing those who had or had not received peer-mediated interventions

Those who had received peer-mediated interventions (peers) had a similar age, marital status, education and income to women who had never received peer interventions (non peers) (data not shown). However, compared with non-peers, peers were more likely to be muslim (42.8% versus 28.3%; *P *= 0.019) and to work from home (24.8% versus 12.5%; *P *= 0.002).

Individuals exposed to peer education had more consistent condom use with clients (86.2% versus 64.0%; *P *< 0.001; Table 2). After adjusting for age, marital status, place of work and education, peers were 2.3 times more likely to suggest condom use (95%CI = 1.0–5.5; *P *= 0.05) and 1.7 times more likely to refuse clients unwilling to use condoms (95%CI = 1.0–2.8; *P *= 0.04). Peers had higher levels of knowledge on HIV and STI than non-peers. Specifically, 79.3% (115/145) of peers compared with 61.2% (221/361) of non-peers knew ≥ 2 symptoms of STI in women, 46.9% (68/145) of peers cited ≥ 2 ways to prevent STI, compared with 23.8% (86/361) of non-peers. These differences were statistically significant (*P *< 0.001).

When comparing STI in peers with non-peers, no significant differences were noted, but all differences were in the same direction. Curable STI (infection with syphilis, gonorrhea or trichomoniasis) were detected in 18.3% (26/142) of FSW who had received peer-mediated interventions, compared with 24.4% (85/348) in those unexposed (*P *= 0.14).

#### Effects of number of peer-education sessions attended

Women who had attended four or more peer education sessions in the past six months reported less sexual partners and higher levels of protected sex than women who attended fewer sessions (Table 3). These women had a lower prevalence of all STI (including HIV), but these differences were not statistically significant. Prevalence of curable STI among those attending only one to three peer education session was 22% (16/73), compared with 14% (10/69) in those attending more sessions (*P *= 0.25). Similar findings were noted when number of peer session attended was analyzed as a continuous variable (data not shown).

**Table 3:** Effects of number of peer education sessions attended on sexual behavior and reproductive tract infections

	**One to three peer education sessions in past six months**	**Four or more peer education sessions in past six months**	AOR** (95%CI)
**UNPROTECTED SEX**			

Total years in sex work, mean (sd)	5.9 (4.4)	6.4 (5.7)	-
≤ 4 sexual partners in past week	53% (39/74)	30% (21/71)	0.4 (0.2–0.9)
≤ 4 one-time clients in past week^§^	34% (23/68)	17% (10/58)	0.4 (0.1–1.0)
Used a condom with last client	89% (66/74)	99% (70/71)	7.2 (0.8–64.4)
Always used a condom with clients	80% (59/74)	93% (66/71)	3.3 (1.0–10.8)
Always used a condom with boyfriend/husband^€^	20% (10/50)	35% (14/40)	1.9 (0.7–5.7)

**SEX WORKER EMPOWERMENT**			

Own idea to use the condom at last sex	92% (61/66)	97% (68/70)	2.9 (0.5–18.4)
Provided the condom at last sex herself	79% (52/66)	76% (53/70)	0.6 (0.3–1.6)
HIV-related knowledge and attitudes			
Thinks a client might be HIV-infected	28% (21/74)	35% (25/71)	1.8 (0.8–4.0)
Knows people with HIV can look healthy	97% (72/74)	97% (69/71)	1.5 (0.1–15.8)
Mentions advantage to knowing HIV status	97% (72/74)	99% (70/71)	0.4 (0.01–11.1)
Knows ≥ 2 symptoms of STI in women	77% (57/74)	82% (58/71)	1.1 (0.4–2.6)
Cited ≥ 2 ways to prevent STI	33% (25/74)	61% (43/71)	3.9 (1.8–8.7)
Ever refused client unwilling to use condoms	85% (63/74)	82% (58/71)	1.2 (0.4–3.3)

**TRANSMISSION EFFICIENCY**			

Awareness of own HIV status	43% (32/74)	46% (33/71)	1.2 (0.5–2.4)
Self reported history of RTI in past year			
Excessive or foul smelling vaginal discharge	47% (35/74)	39% (28/71)	0.7 (0.3–1.4)
Genital ulcer	20% (15/74)	13% (9/71)	0.7 (0.2–1.9)
RTI prevalence			
Syphilis infection	1% (1/73)	0% (0/69)	-
Gonorrhoeal infection	1% (1/73)	0% (0/69)	-
Trichomonas vaginalis infection	19% (14/73)	14% (10/69)	0.8 (0.3–2.3)
Bacterial vaginosis infection	42% (28/66)	44% (28/63)	1.3 (0.6–2.9)
Candida infection	29% (21/73)	23% (16/70)	1.1 (0.5–2.6)

Notes to Table 3:

RTI reproductive-tract infection. Cells contain %, n/N unless indicated.

^Sexual partners are classified: boyfriend/husband, regular clients and one-time clients

^§^Among those women having had a one-time client in the past week

^€^Among those women who reported having a boyfriend/husband

**AOR, odds ratio adjusted for age, marital status, education and place of work. Logistic regression was used to calculate AOR.

### HIV prevalence

When comparing HIV prevalence in 2000 and 2005, a non-significant increase was noted: 30.6% (151/493) versus 33.3% (166/498; *P *= 0.36). No difference was seen among younger women 15–19 years (15%, 4/27 versus 15%, 4/26; *P *= 0.95). HIV infection increased with age to a peak in women 25–29 years in 2000 and then declined with increasing age. In 2005, this peak occurred in older women, 35–39 years. Though not significantly different, HIV prevalence was lower in peers (29.6%; 42/142) compared with non-peers (34.8%; 124/356; *P *= 0.26). HIV prevalence was 25% (17/69) in FSW who attended four or more peer-education session, compared with 34% (25/73) in those attending one to three sessions (*P *= 0.21).

## Discussion

The study aimed to evaluate overall and individual-level effects of five years of peer-mediated interventions among FSW, using a standard framework of outcome measures [5]. Societal changes over time and the presence of other HIV prevention initiatives make it difficult to ascertain the proportion of change attributable to the peer intervention. More can be drawn from the differences noted between women who received peer-mediated interventions and those who had not. Further, behavioral changes were more marked in women with increased exposure to peer education, providing additional supportive evidence for the effectiveness of these interventions. These findings are encouraging and consistent with previous studies which evaluated the ability of peer-mediated interventions to facilitate behavior change among high-risk groups [21,3,23], (though not all) [24].

### Effect on HIV prevalence

Though the burden of HIV in the general population in Kenya has declined in recent years, it is not known whether such changes are mirrored among FSW and other high-risk populations [1,25]. Complex behavioral changes occurred between the two periods and, disappointingly, HIV prevalence remains high in this population, even among younger age groups (a useful proxy for recent infection). Improved survival with wider access to antiretroviral treatment and care may make a minor contribution to this finding. Several other studies have also shown weak correlation between behavioral changes and HIV risk [9,24,26]. Most notably, a community-randomized trial in Uganda found that changes in behavior and an STI treatment intervention did not reduce HIV incidence [26]. Interestingly, FSW who attended peer-education sessions more frequently did show a trend towards lower HIV prevalence, though study design, sample size and potentially confounding factors limit the ability to draw firm conclusions.

### Behavior changes

Condom use during last sex act was about seven times more likely in 2005. Consistent use with clients increased to 70%, similar or higher than seen in other studies in Africa, but, as elsewhere, remained disappointingly low with boyfriends and husbands [27,12,13]. Increased condom use among FSW has been shown to reduce HIV prevalence and incidence [14,21,22,27,28]. However, the best marker of risk for less infectious STI such as HIV, is the total number of unprotected sex acts [29]. Messages about reducing the number of sexual partners were not effective. This is likely due to the close link between number of partners and income, and women have limited alternative sources of income to replace that lost from a reduction in partner number. Overall, it is uncertain whether the balance between higher number of partners and increased condom use reduced the total number of unprotected sex acts.

Women showed stronger condom negotiation skills with high levels of initiation and provision of condoms. An increase in self determination is also suggested as women in 2005 were five times as likely to refuse clients unwilling to use condoms as those in 2000. These are proxy indicators of FSW empowerment at an individual level, while changes in social attitudes and legal status as well as collective associations or networks are needed for more comprehensive empowerment of FSW [5,30] However, for those FSW who use condoms inconsistently, client's refusal remains the most common reason, suggesting that a subset of FSW remains particularly vulnerable. Importantly, only a minority of clients initiated condom use, again highlighting the need for interventions targeting clients of sex workers and the contexts in which they work [31].

Compared with the first time period, women had favorable attitudes towards HIV testing. The marked increase in women knowing their HIV status is an important finding in this study, though some women reported being fearful of HIV testing during interviewing.

### Study limitations

The study design and the absence of prospective controls limit the ability to quantify the effectiveness of the intervention. This is particularly important since marked changes occurred in the study population, possibly resulting from an expansion in tourism and number of bars, with concomitant FSW in-migration. Many new women are likely to have entered female sex work and women reached by peer education left. Over half (278/502) of study participants in 2005 only started practicing sex work in the last five years. An especially notable change over the five years is the decline in number of FSW who are married or cohabiting, which warrants further investigation. Though snowball sampling is appropriate for locating difficult-to-reach populations, this non-random sampling may have contributed to differences noted. The sample size was intended to mitigate such effects.

Over the study period, other interventions have promoted sexual behavior change such as mass-media education, increased availability of widely-promoted VCT services and an HIV prevention study in 2004 which introduced the female condom[13] These concurrent interventions are likely to have impacted on self-reported knowledge of HIV status, the desire to know once HIV status and possibly also on condom use. A further limitation is that many outcomes were self-reported, subject to social-desirability bias [26,32,33]. Nevertheless, observational designs like controlled before-and-after studies can provide strong plausibility support that positive impact has occurred, and highlight aspects that require further development [34,35].

### Policy recommendations

Study findings will be used to inform design of future peer-mediated interventions in this population, with an increased focus on building negotiation skills for women whose clients refuse condom use, strengthening efforts to promote condom use with emotional partners, and early recognition and treatment of STI. Further interventions are required to impact on the gender norms in this setting and to reduce economic dependency. Such interventions were not the focus of this study, which predominately addressed biomedical risk factors. Overall effectiveness may be improved by engaging peer educators from subgroups which had low levels of peer-education coverage, as well as by using qualitative research methods to inform further modification of the intervention [7].

## Conclusion

About 90% of peer educators were retained over five years and were able to reach nearly one third of the target population. These levels of peer educator retention are high, possibly due to rigorous selection procedures and ongoing supportive supervision for their work. Though uncertainty of the effects of the intervention remains, study findings suggest that peer-mediated interventions can change sexual behavior in FSW, but this did not lower the HIV prevalence among FSW, even among younger age groups. Promising results were achieved among FSW who attended peer sessions more frequently, suggesting that effectiveness of peer education is related to intensity of the intervention. Of note, the lack of impact of these peer sessions on condom use among relationships of FSW with boyfriends and husbands remains a major concern. These results also suggest that additional strategies are needed to improve coverage and impact of peer interventions. While peer-mediated strategies remain important for reaching vulnerable groups and improving their self-efficacy, additional HIV prevention technologies and a reduction in socio-economic vulnerability are urgently needed to increase their ability to control risk for HIV acquisition.

## Competing interests

The authors declare that they have no competing interests.

## Authors' contributions

The study was conceived and designed by SL, PM, NK and SW. SL, NK, AR, MB, KM and WB made substantial contributions to acquisition of data. SL analysed study data and drafted the article. MC assisted with the data analysis and revised the article critically for important intellectual content. MT provided overall supervision and revised the article for important intellectual content. All authors assisted in interpretation of data and they read and approved the final manuscript.

## Pre-publication history

The pre-publication history for this paper can be accessed here:


